# Emerging Food Packaging Applications of Cellulose Nanocomposites: A Review

**DOI:** 10.3390/polym14194025

**Published:** 2022-09-26

**Authors:** Jingwen Li, Feifan Zhang, Yaqi Zhong, Yadong Zhao, Pingping Gao, Fang Tian, Xianhui Zhang, Rusen Zhou, Patrick J. Cullen

**Affiliations:** 1 School of Food and Pharmacy, Zhejiang Ocean University, Zhoushan 316022, China; 2 School of Engineering Sciences in Chemistry, Biotechnology and Health, KTH Royal Institute of Technology, 10044 Stockholm, Sweden; 3 Fujian Provincial Key Laboratory of Plasma and Magnetic Resonance, Institute of Electromagnetics and Acoustics, Xiamen University, Xiamen 361005, China; 4 School of Chemical and Biomolecular Engineering, The University of Sydney, Sydney, NSW 2006, Australia

**Keywords:** nanocellulose, nanocomposites, food packaging, fabrication strategies, performance, commercialization

## Abstract

Cellulose is the most abundant biopolymer on Earth, which is synthesized by plants, bacteria, and animals, with source-dependent properties. Cellulose containing β-1,4-linked D-glucoses further assembles into hierarchical structures in microfibrils, which can be processed to nanocellulose with length or width in the nanoscale after a variety of pretreatments including enzymatic hydrolysis, TEMPO-oxidation, and carboxymethylation. Nanocellulose can be mainly categorized into cellulose nanocrystal (CNC) produced by acid hydrolysis, cellulose nanofibrils (CNF) prepared by refining, homogenization, microfluidization, sonification, ball milling, and the aqueous counter collision (ACC) method, and bacterial cellulose (BC) biosynthesized by the Acetobacter species. Due to nontoxicity, good biodegradability and biocompatibility, high aspect ratio, low thermal expansion coefficient, excellent mechanical strength, and unique optical properties, nanocellulose is utilized to develop various cellulose nanocomposites through solution casting, Layer-by-Layer (LBL) assembly, extrusion, coating, gel-forming, spray drying, electrostatic spinning, adsorption, nanoemulsion, and other techniques, and has been widely used as food packaging material with excellent barrier and mechanical properties, antibacterial activity, and stimuli-responsive performance to improve the food quality and shelf life. Under the driving force of the increasing green food packaging market, nanocellulose production has gradually developed from lab-scale to pilot- or even industrial-scale, mainly in Europe, Africa, and Asia, though developing cost-effective preparation techniques and precisely tuning the physicochemical properties are key to the commercialization. We expect this review to summarise the recent literature in the nanocellulose-based food packaging field and provide the readers with the state-of-the-art of this research area.

## 1. Introduction

Food packaging is vital to the food industry, which could protect the food from physical damage, chemical hazards, and biological contamination [1]. Synthetic polymer-based plastics [2], paper and paperboard [3], glass [4], and metal [5] are the most widely used packaging materials, and the choice of the packaging material is food-type-dependent. Nowadays, plastics derived from fossil resources are the most common food packaging materials. By far the most plastic, almost 40%, is used for packaging. Annually, approximately 500 billion plastic bags are used worldwide. After use, plastic products are generally incinerated or landfilled, causing serious environmental pollution. In addition, recycling and processing plastic packaging waste is estimated to cause an annual loss of USD 80–120 billion globally [6]. Today, with the promotion of “sustainable development”, new functional food packaging materials that are green, environmentally friendly, biodegradable, renewable, and sustainable have become the cutting-edge research direction in the food packaging field.

Cellulose is the most abundant biopolymer on Earth. It can be synthesized by trees [7], plants [8], sea animals (tunicates) [9], algae [10], and certain cellulose-secreting bacteria [11], and the properties of the cellulose are also found to be source-dependent [12]. However, cellulose derived from trees is still the most commonly investigated due to its high abundance and easy availability. In fact, paper made from woody cellulose has been used as packaging material for decades, though its low wet strength and poor barrier properties inhibit its further applications as packaging material to replace plastics [13]. More recently, cellulose fibers have been processed to nanoparticles through many different methods, such as acid hydrolysis [14], homogenization [15], grinding [16], blending [17], etc., which are normally terminated as nanocellulose [18]. Though the nomenclature of nanocellulose is still not yet standardized, it is commonly categorized into three types in terms of the preparation method and the morphological profile: CNC (cellulose nanocrystals), CNF (cellulose nanofibrils), and BC (bacterial cellulose) [19]. CNC with a rod-like shape with a width of 3–5 nm and a length between 50–500 nm is typically produced by mineral acid hydrolysis of lignocellulosic materials [20]. Another type of nanocellulose is CNF, with a width of 4–20 nm and a length between 500 nm and a few micrometers. CNF is typically produced via a mechanical treatment of lignocellulosic materials in a high-pressure homogenizer or a microfluidizer, achieving defibrillation and breakage of fibrils. Normally, a complementary pretreatment such as enzymatic treatment [21], carboxymethylation [22], or 2,2,6,6-Tetramethylpiperidine-1-oxyl (TEMPO) oxidation [23] is employed to facilitate the defibrillation, thus lowering the energy consumption [24]. BC is biosynthesized by specific bacteria, with many characteristics differing from CNC and CNF. BC is composed of ribbon-like fibers, with a width in the range of 0.01–0.10 μm and a length between one hundred nanometers to the micron level, and the fibers interweave to form a 3D network [25]. For all these three categories of nanocellulose, they generally have a high degree of polymerization (DP), high crystallinity, good thermal stability, and excellent mechanical properties, making nanocellulose a most promising candidate to develop bio-based nanocomposites for green packaging applications.

In this review, the sources, extraction, and properties of nanocellulose are summarized, and the potential of nanocellulose as the matrix, nanofiller, or coating materials to prepare advanced cellulose nanocomposites for food packaging is discussed: (i) the source-specific physicochemical properties of nanocellulose prepared by various cellulose sources, (ii) the inter-relation between preparation methods and the properties of the obtained nanocellulose, (iii) the fabrication strategies of cellulose nanocomposites aiming for potential food packaging applications, (iv) the performance of the thus-prepared cellulose nanocomposites as packaging materials, and (v) the state-of-the-art in the commercialization of nanocellulose on the market. We also discuss the opportunities and challenges of developing cellulose nanocomposites-based food packaging materials.

## 2. Source and Structure of Cellulose and Its Derived Nanocellulose

**Figure 1:**
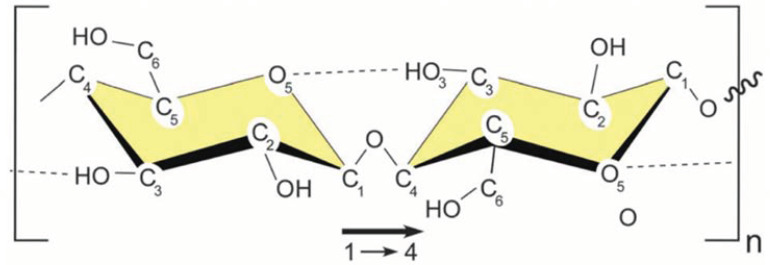
Schematic illustration of cellulose molecule, reprinted with permission from [26], 2011, RSC.

As one of the most widely distributed and abundantly available biopolymer materials in nature, cellulose has been widely used throughout human beings’ history. Cellulose is a linear natural biopolymer composed of β-D-glucopyranose, which has a chemical structure with repeating units of cellulose disaccharide (Figure 1) [26]. The repeating unit is then linked by a β-1,4-glycosidic bond by forming an acetal functional group between hydroxyl groups at the C_4_ position of the glucose unit and a hydroxyl group at the C_1_ position of the adjacent unit. The structural formula of cellulose is (C_6_H_10_O_5_)_n_, where n is the degree of polymerization (DP). There are hydroxyl groups (-OH) at the C_2_, C_3_, and C_6_ positions of each glucose unit [27]. The presence of these -OH groups render the cellulose a high chemical reactivity, which allows a series of chemical and physical modification of cellulose, so that the modified cellulose has the expected properties to meet the different needs for use.

The large number of -OH groups in the molecular structure of cellulose make it easy to form intramolecular and intermolecular hydrogen bonds, leading to the aggregation of cellulose molecular chains and the formation of crystalline supramolecular structure. The supramolecular structure of cellulose is divided into crystalline and amorphous regions [28]. In the crystalline region, many intermolecular hydrogen bonds exist, and the molecular chains are orderly packed. However, molecular interaction is weaker in the amorphous region than in the crystalline region, and the molecular chain arrangement is disordered with a loose structure. The XRD analysis of the crystalline structure of cellulose shows that the intensity of the X-ray diffraction peak in the crystalline region is high, while no specific diffraction peak is observed in the amorphous region [29].

**Figure 2:**
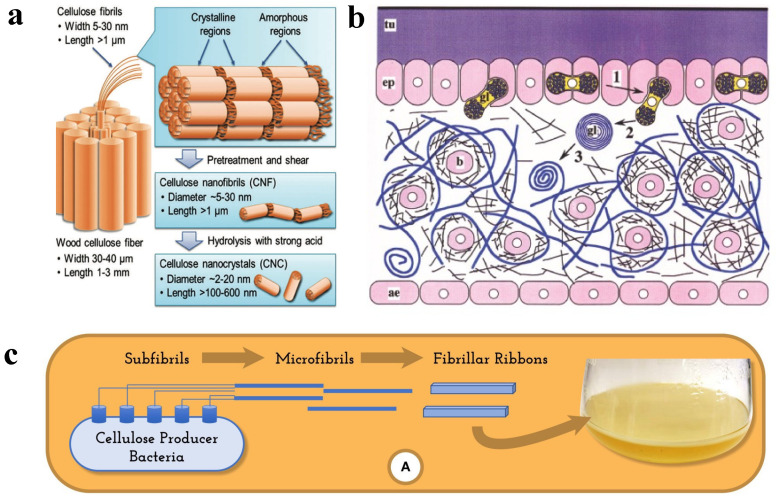
Biosynthesis of (**a**) wood cellulose, reprinted with permission from [35], 2016, ACS; (**b**) tunicate cellulose, reprinted with permission from [36], 2007, Springer; and (**c**) BC, reprinted with permission from [37], 2019, Frontiers Media S.A.

**Figure 3:**
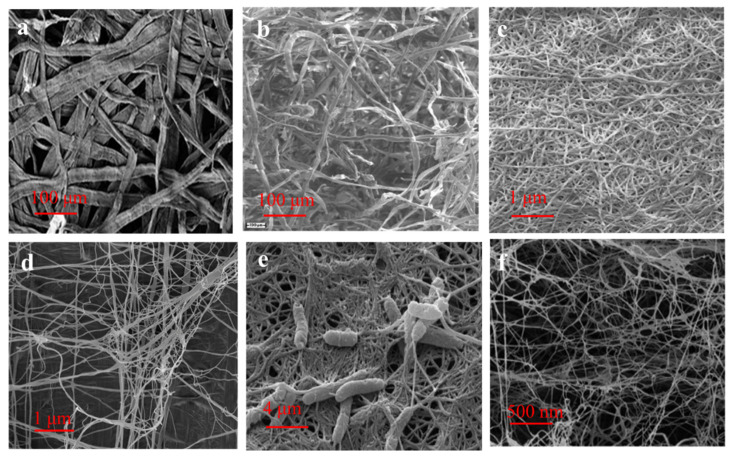
SEM images of cellulose obtained from different sources: (**a**) softwood, reprinted with permission from [43], 2012, RSC; (**b**) cotton, reprinted with permission from [44], 2013, Elsevier; (**c**) tunicate, reprinted with permission from [45], 2015, Elsevier; (**d**) algae, reprinted with permission from [46], 2015, Springer; BC, (**e**) reprinted with permission from [47], 2016, RSC, and (**f**), reprinted with permission from [48], 2022, Elsevier.

In nature, cellulose can be found from many different sources, though the most common ones include wood, plants (cotton, wheat straw, sugarcane bagasse, ramie, hemp, flax, sugar beet pulp, etc.), tunicate, algae, and bacteria [30]. The synthetic mechanism and the properties of the cellulose are source-specific, shown in Figure 2, and the typical microscopic images of cellulose from different sources are presented in Figure 3.

### 2.1. Cellulose Source and Structure

#### 2.1.1. Wood

Trees are the major source of cellulose in nature, in which the cellulose is synthesized through photosynthesis by cells (Figure 2a) [31]. As shown in Figure 3a, the woody cellulose fibers in nature are ribbon-like. Traditionally, the wood is subjected to a pulping process to obtain pure cellulose, which is then further processed into other materials for food, biomedical, environmental mediation, electronic devices, energy conversion, and other applications. In general, woody cellulose-based materials are easily available, renewable, biocompatible, and biodegradable, thus making them ideal raw materials for many advanced biomaterials. Woody cellulose fiber shows excellent physical properties and chemical stability and is a new material for a sustainable society and industrial ecological development [32].

#### 2.1.2. Plant

Cellulose is widely present in most plants, having an annual production of 10^11^–10^12^ tons through photosynthesis, similar to trees. It can be obtained from many sources, mainly cotton, wheat straw, sugarcane bagasse, ramie, hemp, flax, etc. [33]. Plant fibers mainly comprise polymers such as cellulose, hemicellulose, lignin, wax, and pectin. Cellulose is the main component of plant fibers, composed of spirally entangled cellulose microfibrils bound together by an amorphous lignin matrix, for example, cotton cellulose, in Figure 3b. Lignin helps the plants protect against biological attack and acts as a reinforcing agent to strengthen them against gravity and wind, and hemicellulose acts as an adhesive between cellulose and lignin [34]. Plant species, climate, maturity, and soil conditions would affect the physical and chemical properties of plant fibers. Plant fibers are renewable, degradable, low cost, and widely available compared to resources such as oil, natural gas, and coal. Using plant fibers instead of nonrenewable resources is significant in alleviating the energy crisis and environmental pollution problems.

#### 2.1.3. Tunicate

Tunicates are the only animals that can produce cellulose in nature. The tunicate cellulose is generated through a cellulose synthase complex (Figure 2b), and the produced tunicate cellulose is characterized by a large diameter, high crystallinity, and great molecular weight (Figure 3c). Tunicate cellulose is embedded in a protein matrix to form a leathery mantle that can protect the animals from physical damage and predators. It has been reported that there are over 2300 species of tunicates worldwide, and tunicate cellulose has become a new type of biopolymer for many different applications [26].

#### 2.1.4. Algae

Algae can also produce cellulose microfibers in their cell walls, such as green algae, red algae, and yellow-green algae [38]. It has been found that cellulose synthesis occurs at the plasma membrane-bound cellulose synthase, except for some algae that produce cellulosic scales in the Golgi apparatus. There are also great differences in microfiber structure between different algae, probably due to different biosynthesis processes (Figure 3d).

#### 2.1.5. Bacteria

BC is a natural nanostructured polymeric material mainly produced by bacteria [39]. Like tunicate, the bacteria produce cellulose via cellulose synthase complexes (Figure 2c). BC is ribbon-like, with a width in the range of 0.01–0.10 μm, which is 2–3 orders of magnitude smaller than the diameter of plant cellulose (generally 10 μm), and the fiber length ranges from a few hundred nanometers to the micron level, and the fibers cross each other to form a mesh-like structure (Figure 3e,f). BC differs from plant cellulose in that it is not a structural component of the cell wall and therefore does not contain impurities such as hemicellulose and lignin but is a product of microbial metabolism [40,41]. BC not only has the properties of plant cellulose, but also has other, more outstanding advantages, such as high purity, high degree of polymerization, great crystallinity, high hydrophilicity, high permeability and air permeability, excellent Young’s modulus, good biocompatibility, etc. [42]. Under specific culture conditions, BC can be prepared by static fermentation, dynamic fermentation, and fermentation in special molds, showing different structural and performance characteristics.

### 2.2. Nanocellulose Obtained from Different Sources

As mentioned above, cellulose can be extracted from many different sources, and the cellulose properties are source-dependent. After further processing to nanocellulose, the differences are still present in the final products. In Table 1, we summarized the typical geometrical characteristics and crystallinity index of nanocellulose originating from different cellulose sources. A more detailed discussion of the properties of various nanocellulose derived from different sources can be found in Section 3.

## 3. Preparation and Characteristics of Nanocellulose

Nanocellulose can be categorized into CNC, CNF, and BC, wherein CNC and CNF are nanocellulose prepared from biomass, and BC is nanocellulose prepared by microorganisms. The preparation of nanocellulose from biomass is mainly achieved by breaking down the cellulose fibers into nanoscale particles, while BC is a microbially produced cellulose on the nanoscale. Some aerobic nonpathogenic bacteria (for example, Taonella mepensis, Aerobacter, Azotobacter, Rhizobium) can produce BC in the form of exopolysaccharides at the gas–liquid interface, and BC combines the properties of both cellulose and nanomaterials [52]. The pretreatment and extraction techniques used to prepare nanocellulose must be carried out under specific conditions to improve the yield and obtain the desired specifications and properties [53]. Methods for producing nanocellulose vary by source and end application purpose. Table 2 summarizes the nanocelluloses prepared by different methods and their properties.

### 3.1. Pretreatment of Cellulose Fibers for Nanocellulose Production from Biomass

**Figure 4:**
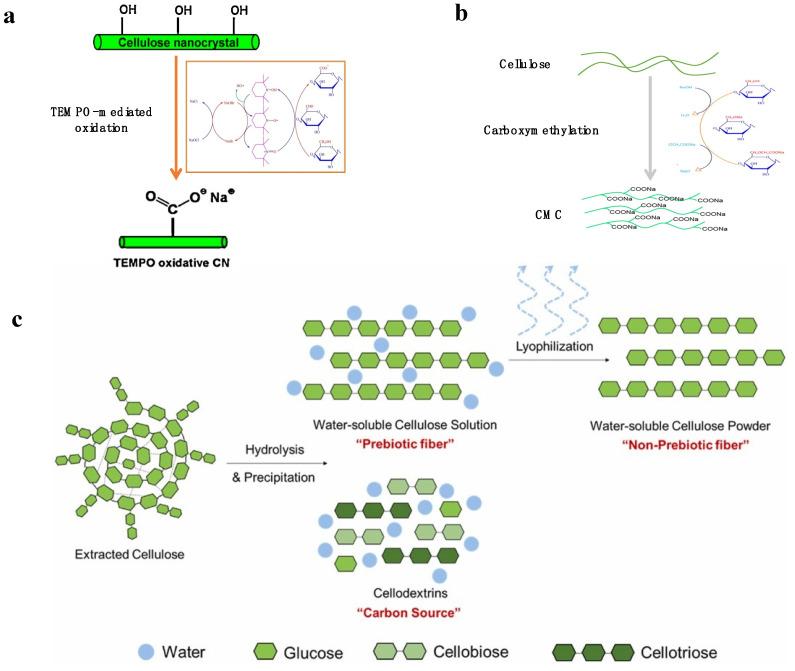
Different pretreatment methods for nanocellulose preparation: (**a**) TEMPO oxidation, reprinted with permission from [69], 2019, Academic Press Inc and [70], 2013, Elsevier; (**b**) carboxymethylation, reprinted with permission from [71], 2022, Elsevier; (**c**) enzymatic hydrolysis, reprinted with permission from [72], 2021, Elsevier.

Appropriate pretreatment methods can change the properties of cellulose and endow the final nanocellulose with better properties. The pretreatment for the production of biomass nanocellulose mainly includes TEMPO oxidation, carboxymethylation, and enzymatic hydrolysis (Figure 4) [54]. After the pretreatment, the cellulose is either modified by functional groups or slightly broken down, thus facilitating the following extraction process by reducing energy consumption and uniforming the particle size.

**Table 2:** Preparation and properties of nanocellulose produced by different methods.

Extraction Method	Cellulose	Diameter (nm)	Length (nm)	Crystallinity Index (%)	Degradation Onset Temperature (°C)	Tensile Strength (MPa)	References
H_2_SO_4_ hydrolysis	Microcrystalline cellulose	10.8 ± 2.4	111.2 ± 25.6	/	270	114	[55]
HCl hydrolysis	Bleached kraft pulp	28.5	481	88.2	310	/	[56]
H_2_SO_4_ hydrolysis and sonicated	Industrial pepper waste (*Piper nigrum* L.)	33.4 ± 11.7	/	69.9	300.6	/	[57]
HCl hydrolysis and sonication		50.7 ± 9.6	/	73.7	291.5		
H_3_PO_4_ hydrolysis, sonicated		67.8 ± 3.1	/	75.8	298.3		
Oxalic acid hydrolysis and sonicated		21.7 ± 4.9	/	77.8	311.2		
Citric acid hydrolysis and sonicated		23.2 ± 0.6	258.8 ± 58.4	76.4	310.1		
Acetic acid hydrolysis and sonicated		48.7 ± 9.4	343.7 ± 2.3	78.3	308.2		
Alkaline treatment and blending	Oil palm empty fruit bunch	89	/	/	/	33.0	[58]
Disc grinder	Raw wood	5 ± 3	/	67	235	233	[59]
Ball mill	Raw sisal	12.35	/	53.6%	/	92.73	[60]
Ultrafine grinder	Unbleached Eucalyptus kraft pulp	38 ± 16	3000	/	/	/	[61]
High pressure homogenization	Cellulose powder (cotton linters)	46.4 ± 7.5	417.7 ± 37.6	/	270	114	[55]
Homogenizer and sonication	BC (*K. oboediens R37-9*)	6.06 ± 0.96	815 ± 0.95	75.64	/	142	[62]
High pressure homogenization	Bleached softwood kraft pulp board	18.85 ± 4.51	/	67.3	/	177.99	[63]
Grinder	Hardwood bleached kraft pulp	5.5 ± 1.6	/	/	/	/	[64]

#### 3.1.1. TEMPO Oxidation

**Figure 5:**
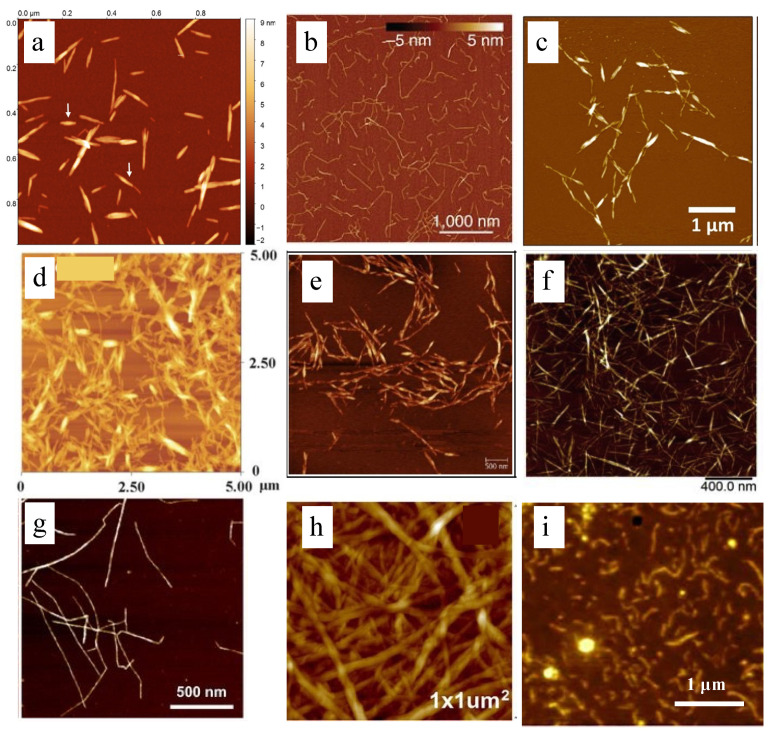
Nanocellulose prepared from different methods: (**a**) H_2_SO_4_, reprinted with permission from [81], 2021, Springer; (**b**) TEMPO, reprinted with permission from [82], 2015, nature; (**c**) H_3_PO_4_, reprinted with permission from [83], 2019, Springer; (**d**) HCl, reprinted with permission from [84], 2013, RSC; (**e**) maleic acid, reprinted with permission from [85], 2017, Wiley; (**f**) oxalic acid, reprinted with permission from [86], 2019, Elsevier; (**g**) TEMPO-oxidized CNF, reprinted with permission from [87], 2021, Springer; (**h**) enzymatic hydrolysis, reprinted with permission from [88], 2017, Springer. (**i**) carboxymethylation, reprinted with permission from [71], 2022, Elsevier.

2,2,6,6-Tetramethylpiperidin-1-oxyl (TEMPO) is a water-soluble piperidine nitroxide radical. TEMPO oxidation method uses sodium hypochlorite (NaClO) as the main oxidant and TEMPO and sodium bromide (NaBr) as the catalyst, thus forming TEMPO/NaBr/NaClO as a new selective oxidation system, which can be used for the oxidation of cellulose (Figure 4a). Under specific conditions (pH 9~11), the primary hydroxyl group at the C_6_ position of cellulose can be selectively oxidized to the carboxyl group without changing the fiber morphology and crystallinity, thus improving the colloidal stability of the final CNF [65]. This suggests that carboxylate groups formed by TEMPO oxidation are selectively introduced into the surface of cellulose nanofibers, rather than the inner cellulose crystallites [66]. TEMPO-oxidized CNF has long and highly flexible network structures that are more uniform and better dispersed in the aqueous phase (Figure 5b,g) [67]. Kaffashsaie et al. used wood as a raw material to prepare nanocellulose through TEMPO oxidation. The dispersion is stable after even more than 72 h, which was better than the nanocellulose prepared directly by mechanical refining. This is due to the electrostatic repulsion of carboxylate ions among nanocellulose [59]. TEMPO oxidation is normally carried out in a mild environment, and nanocellulose can be obtained by simple mechanical treatment of cellulose after the reaction is completed, which greatly avoids the disadvantage of consuming more energy by simply using mechanical treatment to obtain nanocellulose. Unfortunately, the thermal stability of nanocellulose will be reduced due to the introduction of -COOH groups on C_6_ of cellulose nanofibers [68].

#### 3.1.2. Carboxymethylation

Carboxymethylation is considered to be another commonly used pretreatment method. Firstly, the cellulose was swollen in a mixture of aqueous sodium hydroxide, and then an organic solvent (usually, alcohols, such as isopropanol) was added under vigorous stirring. After that, chloroacetic acid (CAA) or sodium chloroacetate (SCA) is slowly added for carboxymethylation. The carboxymethylation reaction is based on the Williamson ether synthesis mechanism, in which the 1° and/or 2° hydroxyl (-OH) of cellulose can be etherified with carboxymethyl. The principle of carboxymethylation is to form more nucleophilic alkoxide groups after deprotonating the hydroxyl group (-OH) of polysaccharides in an alkaline solution. Then, -CH_2_COONa was introduced on cellulose by SN2 reaction between CAA or SCA and cellulose alkoxide (Figure 4b) [73]. Furthermore, by breaking the hydrogen bonds within the cellulose structure, the presence of bases can facilitate or initiate the reaction process and bring about uniform chemical changes in the single cellulose chains (Figure 5i) [74]. The occurrence of side reactions can be avoided under specific conditions by tuning chemical reagent concentration, reaction time, and temperature, thereby improving carboxylated nanocellulose properties and yields [75]. The degree of substitution (DS) of carboxylated nanocellulose is a key factor affecting its properties. The maximum substitution degree of carboxylated nanocellulose is 3, but when the substitution degree of carboxylated nanocellulose is too small (<0.4), carboxylated nanocellulose is not dispersible. In fact, DS increases significantly by increasing reaction temperature and time; however, the time has a greater effect on DS than temperature [76]. Sarmina et al. analyzed the effects of NaOH concentration, monochloroacetic acid (MCA) concentration, time, temperature, and cellulose particle size on DS, optimized the carboxymethylation reaction of cellulose, and prepared nanocellulose with DS up to 2.41 from corn husk [77].

#### 3.1.3. Enzymatic Hydrolysis

Enzymatic hydrolysis is a biological treatment method that involves the digestion or modification of cellulose fibers with enzymes to promote further shortening and defibrillation of the cellulose (Figure 4c). Due to the good selectivity of the enzyme, the extracted nanocellulose does not change much after the enzymatic hydrolysis (Figure 5h) [68]. The main enzymes used for this purpose are cellulases, which break the β-1,4 glycosidic bonds in the cellulose molecule. More specially, the cellulase can only destroy the amorphous regions within the cellulose, thereby reducing the energy requirements for mechanical processing [78]. Dias et al. found that the content of fine particles after enzyme pretreatment increased from 55 ± 1% after laccase treatment to 62 ± 1% after cellulase treatment. These results showed that cellulase could expand the fiber by attacking the surface and interior of the fiber, making more water molecules enter the fiber [61]. However, different cellulases would produce different nanocelluloses. For example, endoglucanases can produce a mixture of CNF and CNC, while exoglucanases can produce nanocellulose complex structures [79]. The enzymatic hydrolysis method employs milder conditions, a promising green and sustainable method, although the reaction time is usually longer. Moreover, the high cost of enzymes is still a persistent issue, but reducing the cost by reusing enzymes by developing enzyme immobilization techniques can potentially solve this problem [80].

### 3.2. Extraction Method of Nanocellulose from Biomass

After decades of intensive investigations, many different nanocellulose extraction methods have been developed and improved. We summarized the recent progress in the method development. In general, the nanocellulose preparation methods can be normally categorized into acid hydrolysis and mechanical treatment, or a combination thereof, and biosynthesis.

#### 3.2.1. Acid Hydrolysis

**Figure 6:**
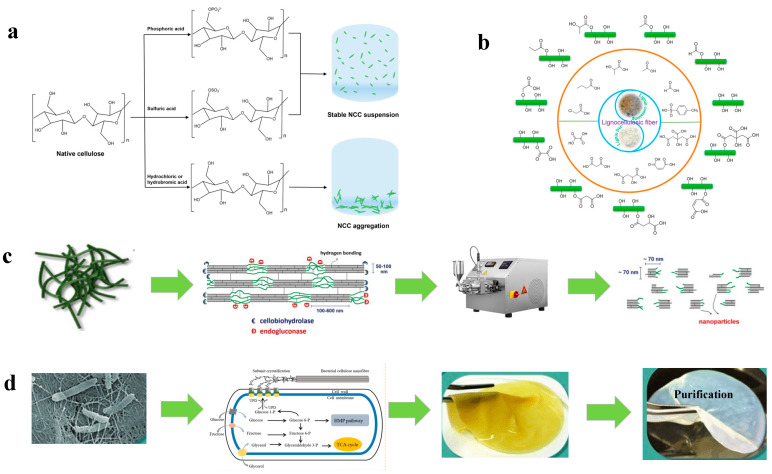
Different preparation methods of nanocellulose (**a**) inorganic acid hydrolysis, reprinted with permission from [98], 2022, Elsevier; (**b**) organic acid hydrolysis, reprinted with permission from [99], 2021, Elsevier; (**c**) mechanical treatment, reprinted with permission from [71], 2022, Elsevier and [100], 2021, Elsevier; (**d**) microbial synthesis, reprinted with permission from [101], 2022, Elsevier and [37], 2019, Frontiers Media S.A.

Acid hydrolysis is the most commonly used method to isolate nanocellulose, in which strong acids easily hydrolyze the amorphous regions of whole cellulose fibers to produce CNC with reduced size. The obtained CNC has a similar morphology to the original cellulose and has a higher degree of crystallinity. Negatively charged nanocelluloses with abundant functional groups can also be produced by acid hydrolysis; for example, the nanocellulose with sulfate groups can be stably dispersed after sulfuric acid hydrolysis aqueous solutions due to surface charge repulsion [89]. The acids used for acid hydrolysis can be divided into inorganic acids (such as sulfuric acid, hydrochloric acid, phosphoric acid, etc.) (Figure 6a) and organic acids (such as oxalic acid, citric acid, maleic acid, etc.) (Figure 6b). After the reaction, the obtained nanocellulose is washed with deionized water, centrifuged to remove residual acid, and then suspended in distilled water. Sometimes, the acid hydrolysis is always followed by a mechanical process (such as high-pressure homogenization, microfluidization, ultrasound, etc.) to disperse the CNC into a uniform and stable suspension. The properties of the CNC suspension are closely related to the hydrolysis conditions (acid type, concentration, reaction temperature, and time) and the mechanical treatment conditions [90]. The main disadvantage of this process is generating a lot of acid-containing wastewater, which must be treated until discharged into the environment.

##### Inorganic Acid Hydrolysis

By using H_2_SO_4_, uniform and short CNC with narrow polydispersity and high crystallinity of over 90% can be produced, which also shows good colloidal stability (Figure 5a). In addition, during the sulfuric acid hydrolysis, sulfate groups are generated on the surface of nanocellulose, thereby forming a negatively charged layer on the CNC, which further improves the dispersibility of CNC. However, residual sulfate groups (-SO_4_^2−^) can induce dehydration, resulting in lower thermal stability. In addition, when higher hydrolysis temperatures above 45 °C are applied, H_2_SO_4_ hydrolysis not only removes the amorphous region but also partially dissolves the crystalline region, resulting in a size reduction of CNC [91]. Due to the small size of the CNC, this provides a large surface area for heat treatment, which can also lead to a reduction in thermal stability [92]. Worse, H_2_SO_4_ hydrolysis sometimes causes the degradation of cellulose due to its strong oxidizing property, which negatively impacts the large-scale production and application of CNC.

Hydrochloric acid (HCl) hydrolysis is another method used to prepare CNC. Unlike H_2_SO_4_ hydrolysis, HCl hydrolysis provides unmodified and uncharged CNC, which can form more intermolecular hydrogen bonds (Figure 5d) [93]. CNC prepared by HCl hydrolysis can form stable Pickering emulsions, whereas H_2_SO_4_-hydrolyzed CNC does not possess this property [94]. Since the surface of CNC prepared by HCl hydrolysis is not charged, it is easy to flocculate in water and does not produce a stable suspension, but its thermal stability is higher than that prepared by H_2_SO_4_ hydrolysis [95]. Shang et al. used cetyltrimethylammonium bromide (CTAB) to modify the nanocellulose prepared by HCl hydrolysis and found that the stable CNC dispersion was formed by using low-concentration CTAB, and no aggregation occurred after 7 days of storage [56].

Hydrolysis of cellulose by phosphoric acid (H_3_PO_4_) produces CNC with phosphoric acid half-ester groups, so it is slightly charged [96]. This colloid’s stability is much better than that produced by hydrochloric acid hydrolysis, which does not introduce surface charges [20]. The CNC produced by phosphoric acid hydrolysis has a more inhomogeneous profile compared with those prepared by sulfuric acid hydrolysis, so easier flocculation can be expected, but it shows better thermal stability (Figure 5c) [97].

##### Organic Acid Hydrolysis

Organic acids are greener alternatives to inorganic acids for CNC preparation because they are less acidic and corrosive. Since most organic acids have lower boiling points than inorganic acids, they are easier to be recycled. Similar to H_2_SO_4_ and H_3_PO_4_ hydrolysis, the organic acid may also modify the CNC surface, especially when strong inorganic acids (for example, H_2_SO_4_) are present as a mixture. Wang et al. used bleached eucalyptus kraft pulp as raw material and applied a small amount of sulfuric acid (5–10 wt%) to effectively improve the hydrolysis efficiency of formic acid. Under the best reaction conditions, the maximum yield of CNC was 70.65% [102]. The organic acids usually introduce hydrophobic moieties on the surface of CNC, facilitating CNC dispersion in nonpolar organic solvents (Figure 5e,f). The introduction of negatively charged carboxyl groups, for example, by oxalic acid hydrolysis, can improve the stability of CNC suspensions [99].

#### 3.2.2. Mechanical Process

Cellulose fibers can be mechanically processed by different mechanical methods to extract nanocellulose, and the most studied techniques include ultrasonication, ball milling, microfluidization, and high-pressure homogenization (Figure 6c) [53]. Compared with acid hydrolysis, the mechanical process is simple, efficient, and does not require chemical solvents. However, the main disadvantages of these processes are high energy consumption and easy blockage of equipment, though the abovementioned pretreatments can alleviate these problems. Dias et al. prepared CNF using an ultrafine mill after laccase pretreatment. It has been found that compared with CNF prepared directly by mechanical defibrillation without pretreatment, the cellulose fibers delaminated faster and made the microfibrils easier to be dispersed, resulting in a 42% reduction in energy consumption by lowering energy consumption from 10.5 kWh/kg to 6.1 kWh/kg [61].

The extraction of nanocellulose by ultrasonication is a mechanical method based on ultrasonic waves. During ultrasonication, liquid molecules can absorb ultrasonic energy and create a cavitation effect, leading to the formation, expansion, and explosion of microscopic gases. However, this processing generates a lot of heat; therefore, it is usually performed in cooling equipment to control the temperature under the acceptable level [103]. The use of ultrasonic treatment after acid hydrolysis can improve CNC yield and properties. Ultrasonic treatment can also be used in the pretreatment stage of the nanocellulose production process. After sonication, lignocellulosic feedstocks undergo various changes, such as the destruction of cell wall structure, the increase in specific surface area, and the decrease in the DP of the cellulose component [104].

Another mechanical process is homogenization. In this process, the cellulose suspension (about 2%) passes through the tiny gap between the homogenization valve and the impingement ring; the generated high pressure could produce several destructive forces (including cavitation, turbulence, and shear effects). These destructive forces can significantly disrupt the cellulose hydrogen bond network structure, reducing the size of the fibers to the nanometer range, which in turn affects the nanocellulose properties [105].

Microfluidization is another most common mechanical treatment technique, and it works best for the delamination and fibrillation of cellulose fibers [106]. A microfluidizer consisting of a booster pump accelerates the fiber suspension (0.5–2% by mass) under high pressure, going through an ultrathin chamber with a specific geometry (such as a Z-shape or a Y-shape), and the fibers pass through the channel walls. During this process, the strong shear and shock effects fibrillate cellulose to nanoscale dimensions, and these nanoparticles have a uniform diameter of less than 100 nm. Wang et al. used the blended software kraft pulp as the raw material and applied a high-shear fluid microfluidizer to prepare the nanocellulose. The width and length distributions are about 10~40 nm and 600~1000 nm, respectively. The high aspect ratio makes it a potential candidate as a reinforcing material [102].

Ball milling is also a useful technique to prepare nanocellulose, which is a fast, easy-to-operate, and cost-effective method, showing great potential for industrial applications. During this treatment, the cellulose suspension is kept in a hollow cylindrical container partially filled with spherical tools (made of zirconia, ceramic or metal, etc.). Cellulosic fibers are broken down by the energy released in ball-to-ball collisions and the high grinding energy from ball-to-wall friction as the container rotates [103]. When nanocellulose is produced using this process, wet milling is required to maintain the fibrous state and prevent serious defibrillation to an amorphous state [18]. In addition, the shape of the balls, the weight ratio of the balls to the cellulose, the treatment time, and the moisture content are the influencing factors for the preparation of nanocellulose by ball milling [105].

The aqueous counter collision (ACC) method was first developed by Kondo, in which equivalent aqueous suspensions of cellulose are ejected from dual nozzles under high pressure of 200 MPa and collide at high speed to destroy the weak hydrogen bonds in cellulose, thus producing nanocellulose without chemical modification. The number of collisions and the collision pressure are the influencing factors to determine the performance of the obtained nanofibers [107]. More interestingly, compared with other CNFs, CNF prepared by ACC (ACC-CNF) has a relatively hydrophobic surface [108], since ACC could increase the I_α_ Phase transition to a more stable I_β_ Phase, while keeping its crystalline structure unchanged [107]. Yokota et al. further modified the ACC-CNF through acetylation without changing the original nanofiber morphology, which has proven to improve the dispersibility of the nanocellulose in water and stabilize plastic resin particles in water [108]. Ishida et al. prepared a Pickering emulsion by adsorbing ACC-CNF onto the O/W interface, which was then used as a reaction platform for chemical modification to introduced acetyl groups due to the increased surface [109].

### 3.3. Preparation of BC

The performance of BC depends on the composition of the culture medium and the used bacteria species (Table 3). BC is a natural nanostructured polymeric material mainly produced by bacteria. Similar to tunicate, the bacteria produce cellulose via cellulose synthase complexes (Figure 6d). The proper removal of impurities from the BC matrix helps to improve its mechanical properties. However, the production of BC by microbial fermentation requires using nutrient-rich mediums, in which the HS medium is the most-employed one. Recent work on an improved HS-based medium has focused on alternative sources that can provide a carbon source. Huang et al. used molasses instead of glucose as the carbon source in HS medium, and the produced BC shows improved stability, which is not easy to undergo oxidation reaction and physical modification. The BC yield of 6.67 g is obtained at 1.1 g/L molasses, equivalent to 5 g/L glucose [110]. Nascimento et al. cast BC-derived CNC to produce films, and the results showed that 5% concentration could result in a film with significantly improved tensile strength (from 36.9 to 46.5 MPa), increased elongation at break (from 8.06 to 13.5%), and lower water vapor permeability (17%). In addition, the water resistance of the film was improved, while showing great biocompatibility with Caco-2 cells [111]. Nam et al. used BC as a raw material to prepare CNC-containing aldehyde groups at the surface, which was then combined with silk sericin through physical enhancement and chemical crosslinking to prepare composite films. Compared to the control silk sericin film, the prepared composite film showed higher mechanical properties, better UV blocking, and waterproof and antioxidation properties [112].

## 4. Fabrication Strategies of Cellulose Nanocomposites for Food Packaging

Available studies have shown that nanocellulose is renewable and environmentally friendly, making it a promising candidate for preparing biomaterials for food packaging. To meet the market requirements for food packaging materials, such as strength, water, and oil resistance, nanocellulose-based composites have been intensively explored [115]. The widely used production methods of nanocellulose-based composites for potential food packaging applications are summarized in Table 4.

### 4.1. Solution Casting

**Figure 7:**
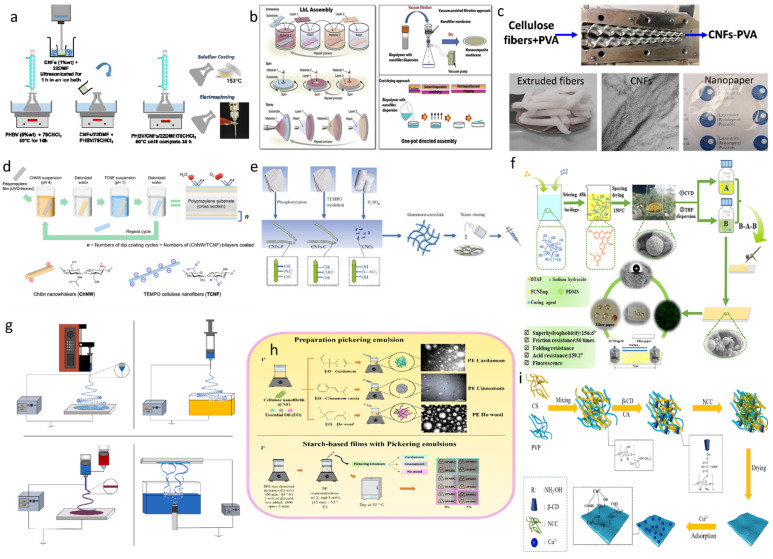
Fabrication strategies of nanocellulose-based composites: (**a**) solution casting, reprinted with permission from [144], 2017, Universidade Federal do Rio de Janeiro—UFRJ; (**b**) Layer-by-layer assembly (LBL), reprinted with permission from [54], 2019, Elsevier; (**c**) extrusion method, reprinted with permission from [145], 2022, Elsevier; (**d**) direct coating method, reprinted with permission from [146], 2021, Elsevier; (**e**) hydrogels, reprinted with permission from [147], 2022, Elsevier; (**f**) spray drying method, reprinted with permission from [148], 2022, Elsevier; (**g**) electrostatic spinning, reprinted with permission from [149], 2022, Elsevier; (**h**) micro-nanotechnology and nanoemulsions, reprinted with permission from [150], 2021, Elsevier; (**i**) adsorption, reprinted with permission from [151], 2022, Elsevier.

One of the easiest ways to make nanocellulose-based packaging materials is solution casting. In this method, two or more solutions are mixed and cast onto a Teflon dish; the following evaporation of the solvent, normally water, will induce the nanocellulose to self-assemble, and a film is then obtained (Figure 7a). Krstić et al. prepared nanocellulose films to encapsulate insoluble drugs by mixing with polyethylene oxide (PEO) polymers through solution-casting. The increased porosity and uniformly sized particles improved the wettability and increased the surface area of the film, making it satisfactory for the controlled release of the drugs [126]. Oldoni et al. extracted nanocellulose from mango pulps, which was further cast in solution to produce a thin, light-weight, highly water vapor permeable and ductile film [127]. By taking advantage of the good film-forming properties of the seaweed-derived alginate and carrageenan, Rajeswari et al. blended nanocellulose with alginate and carrageenan to produce a film with strong mechanical toughness [128].

### 4.2. Layer-by-Layer Assembly

The Layer-by-Layer (LBL) assembly technique was originally proposed by Decher et al. (1997). The mixed solution is organized layer-by-layer through this method to form a thin film. This common technique allows for the facial preparation of films and coatings, which has the advantages of little space requirement and easy operation, thus allowing a wide range of applications (Figure 7b). The LBL technique could produce a wide range of films with different properties because it could take advantage of different polymer characteristics. Koca et al. employed the LBL technique to modify food packaging materials with lysozyme, nanocellulose, and Arabic gum, showing good barrier properties [129]. Yu et al. conducted various studies on the use of LBL technology to prepare composite films for potential food packaging applications, in which nanocellulose, nanosilver, carrageenan, and chitosan were used to achieve the great antimicrobial activity, good breathability, and excellent water barrier properties. The obtained film is nontoxic and effective in inhibiting common pathogenic bacteria [130].

### 4.3. Extrusion

The fabrication of nanocellulose films by extrusion is a more productive and economical method and is mostly used in industrial production. With this technique, the packaging films can be produced on a large scale. Normally, the polymer is extruded through the extruder equipped with a die made of metal in the desired pattern to form the desired shape (Figure 7c). For example, extrusion can be used to produce tubular nanocellulose films [131]. Karkhanis et al. have prepared PLA/CNC nanocomposite films by extrusion. At 25 °C and relative humidity of 50% or more, this laminated film can extend the shelf life of biscuits by more than 40% compared to conventional PLA films [132]. In another study, PLA/CNC nanocomposite films made by extrusion showed good barrier properties against water vapor and oxygen [133].

### 4.4. Direct Coating Method

The coating method involves the application of edible and environmentally friendly natural polymers, which are either dipped or coated directly onto the food surface. These coating substances are commonly based on nanocellulose and other naturally extracted bioactive compounds. Studies have found that the films made of these compounds have good antibacterial properties and better performance in preventing moisture loss and controlling the food oxidation rate (Figure 7d). Using such biopolymers, such as nanocellulose, as a coating material can effectively control the loss rate of its nutrients in the environment. Quintana et al. prepared a chitosan-CNC edible coating material composed of essential oils and bioactive plant extracts and applied it directly to strawberries. The results showed that this coating could effectively inhibit microbial growth and delay fruit spoilage [134].

### 4.5. Hydrogels

Hydrogels are known to be hydrophilic materials in which the polymer molecules are bonded to each other by chemical and physical interactions. Nanocellulose hydrogel has various applications in food packaging (Figure 7e). For example, it has been found to increase the wetness of the food and improve the aesthetics and biodegradability of the food packaging materials. Lu et al. prepared a hydrogel-based freshness indicator using bagasse nanocellulose, which could accurately indicate the degree of discoloration of chicken [135]. Moradi et al. used hydrogel technology to produce a BC-based biofilm that can be used as a new pH-sensing indicator for evaluating the freshness of fish [136]. Pourjavaher et al. developed a food-grade film based on BC nanofibers and extracts of red kale (Brassicaceae), an effective pH indicator at the food contact surface [137].

### 4.6. Spray Drying Method

As the name suggests, the spray drying method sprays the polymer onto the surface of the food by pushing the polymer solution through a nozzle. The sprayed amount of the polymers could be adjusted by controlling the desired shape of the spray nozzle and the concentration of the solution. After the spray drying, a thin film is formed on the food surface for protection (Figure 7f). Throughout this process, the liquid is first conveyed to a peristaltic pump. Due to the force of the compressed air, the liquid is atomized into smaller droplets and sprayed out. In turn, these small droplets are evaporated immediately by the hot air to form dry particles [119]. When this spray drying method is used based on the solution containing CNC and polylactic acid, the air permeability of the food was found to be significantly lowered. Rojas-Lema et al. blended the copper(II) sulfate-decorated chitosan particles and nanocellulose, spray drying was used to coat these substances onto fruit surfaces to extend shelf life [138].

### 4.7. Electrostatic Spinning Technology

Electrostatic spinning technology is a simple, flexible, and operable emerging technology. It is mainly used to create membranes using the electrostatic force-enabled spinning technology. This process facilitates the manufacture of biopolymers, which could include both natural and synthetic polymers, and a wide variety of fiber-shaped polymeric materials can be formed (Figure 7g). Pasaoglu et al. employed electrostatic spinning technology to produce nanocellulose membranes with greater environmental friendliness, sufficient surface functionality, good mechanical properties, and excellent shape stability [139]. Maria Leena et al. used electrostatic spinning to prepare corn protein nanofibers containing nanocellulose and resveratrol, which were effective in controlling water loss of food and maintaining the freshness of apple slices for six hours [140].

### 4.8. Micronanotechnology and Nanoemulsions

Micro- and nanoencapsulation are techniques for encapsulating various bioactive compounds in capsules to protect them from harsh external environments. It is also considered a protective layer for various components (Figure 7h). The functional properties of microcapsules are related to encapsulation efficiency, size, morphology, stability, and release characteristics. This technology involves the formation of polymeric films into small continuous droplets capable of covering solids or liquids. Nanoencapsulation refers to the encapsulation of bioactive substances built on the nano- and microscale. Nano- and microencapsulation is receiving increasing attention as an effective method to improve the bioavailability of bioactive substances because most bioactive agents are unstable in normal environments and deteriorate in contact with water and air [141]. Many available studies have found that using micro- and nanoencapsulation techniques can effectively preserve food products. Salvia-Trujillo et al. had developed a biodegradable nanocellulose-sodium alginate-based film containing lemongrass essential oil, which showed significant antibacterial activity and great browning reaction-inhibiting effects when they were tested to pack fresh-cut Fuji apples, having an extended shelf life of up to two weeks [142]. This environmentally friendly nanocomposite can be used to extend the shelf life of perishable foods.

Nanoemulsions are prepared by adding certain emulsifiers in order to stabilize the oil and water phases, thus producing transparent dispersions with lower viscosity and more stable thermodynamics. As the name suggests, nanoemulsions are emulsion systems with particle sizes between 5 and 100 nanometers and are used specifically for industrial applications, including food, pharmaceuticals, nutritional products, oil extraction, and environmental and agricultural products. Molet-Rodríguez et al. prepared a nanoemulsion containing orange essential oil and nanocellulose, which was employed to stabilize apple juice and showed great bactericidal properties [143]. In general, compared to conventional emulsions, nanoemulsions are normally more effective in inhibiting bacteria.

### 4.9. Adsorption

Adsorption is the process in which a solid material uses its own properties to hold a gas or liquid as an encapsulated film on its surface. Adsorbents incorporated with various polymers, such as nanocellulose and its derivatives, play an important role in improving packaging systems (Figure 7i). Jorge Padrão developed BC films adsorbed with bovine Lactoferrin (BLF) at the surface, which showed a strong bactericidal effect against *E. coli* and *Staphylococcus aureus* [124].

## 5. Performance of Nanocellulose-Based Composites as Food Packaging Materials

Food packaging materials are an extremely important part of the food processing industry and have always been the research focus in the food field. As ideal food packaging materials, they should protect commodities, maintain food quality stability, increase commercial food value, promote sales, and facilitate storage and logistics [152]. Non-biodegradable polymers derived from fossil fuels are the most used materials in food packaging. With increased focus on global environmental issues, the development of biodegradable polymer materials has gained great interest. In recent decades, nanocellulose has been predominantly employed to create biocomposites because of its green source, high specific surface area, high crystallinity, and nontoxic and biodegradable qualities. Many research works have proven that combining nanocellulose and other substances may provide beneficial functionalities, such as barrier characteristics, mechanical properties, antibacterial properties, etc. [153]. The application and advantages of cellulose nanocomposites in the food packaging are summarized in Table 5.

### 5.1. Barrier and Mechanical Properties

For ideal food packaging materials, barrier and mechanical properties are critical to realistic applications. On the one hand, good barrier properties could protect the food from gas and moisture and slow the oxidation reaction and spoilage; on the other hand, the excellent mechanical properties would avoid physical and chemical damage during transportation and sales.

Shi et al. successfully developed cellulose-based food wrapping paper with high barrier and antibacterial properties by constantly depositing multilayer films on the surface of the paper using chitosan (CS) and carboxymethylated nanocellulose. The obtained multilayer coating not only enhanced the paper’s resistance to grease, oil, water, air, and water vapor, but also improved the paper’s mechanical strength. The modified wrapping paper exhibited no visible cytotoxicity and had an antibacterial rate of 95.8% against E. coli and 98.9% against Staphylococcus aureus [154].

CNC and garlic extract (GE) from garlic peel were blended with chitosan to prepare biocomposite films. UV barrier, thermal and mechanical properties, biodegradability, and antibacterial activities were tested on the films. CNC enhanced tensile strength, Young’s modulus, and elongation, compared to chitosan films, but decreased film transparency. On the other hand, the combination of CNC and GE slightly lowered the mechanical properties. The inclusion of CNC reduced the transparency of the film marginally, whereas the addition of GE dramatically increased the UV barrier properties. The integration of CNC and GE did not influence the thermal stability of the chitosan films. The chitosan composite films’ degradability rate was greater than that of neat chitosan films. The antibacterial characteristics of films were investigated against *E. coli*, *Streptomyces griseorubens*, *Streptomyces alboviridis*, and *Staphylococcus aureus*, which found that GE in composite films significantly inhibited bacterial growth. Due to the improved physical properties and better antibacterial activity, chitosan films containing both CNC and GE from garlic peel showed potential as active food packaging materials [155].

Translucent films were made from faba bean protein isolate (FBP) using the solution casting method, reinforced with varied CNC content (1, 3, 5, and 7 wt%) prepared by acid hydrolysis of pinecones, while glycerol was used as a plasticizer. The FTIR and SEM data confirmed that intramolecular interactions between CNC and proteins could induce a more compact and uniform film. These interactions had a favorable impact on mechanical strength, as seen by greater tensile strength and Young’s modulus compared to control films, though much stiffer films were expected as the CNC content increased. The addition of CNC increased the thermal stability of the FBP films by raising the typical onset degradation temperature. Furthermore, the linkages formed between CNC and proteins reduced the water affinity of the films, resulting in a decrease in moisture content and water solubility as well as an increase in water contact angle, resulting in more hydrophobic films as the CNC content in the matrix increased [163].

CNC modified by TEMPO oxidation (TM-CNC) was used to improve the performance of canola protein-based films. Varied weight ratios of modified (TM-CNC) and unmodified nanocrystalline cellulose (U-CNC) were tested. 19.61% of initial -OH groups were transformed to -COOH groups by TEMPO oxidation. The addition of U-NCC and TM-NCC boosted tensile strength substantially, with the greatest value of 8.36 MPa for 5% TM-NCC, compared to 3.43 ± 0.66 MPa for control films. In contrast to the control, both U-NCC and TM-NCC improved the water barrier and thermal characteristics of the films [164].

Cellulose nanofibrils-CNF with less than 1% of lignin and lignocellulose nanofibrils-LCNF with 16% of lignin were blended in various ratios to prepare composite films. The inclusion of LCNF in the formulations increased the films’ antioxidant and UV-blocking characteristics, as well as their mechanical and barrier properties. The addition of 25% LCNF to CNF films improved mechanical properties (94% increase in tensile stress and 414% increase in tensile strain at break) while lowering the water vapor transmission rate by 16% and oxygen transmission rate by 53%. The presence of nanocelluloses with varied chemical compositions and morphologies have contributed to the improved performance. Moreover, the presence of lignin in LCNF helped to increase interfacial adhesion between CNF and avoid the formation of accessible pathways for gas molecules [165].

### 5.2. Antibacterial Property

Food rich in nutrients and water is prone to microbial spoilage, thus leading to great economic loss. In order to prevent that, food packaging materials with satisfactory antibacterial properties are always necessary.

Thongsrikhem et al. used cinnamaldehyde as a crosslinking agent and an antibacterial ingredient to make a gelatin-BC nanocomposite membrane (GCB). Heat treatment at 120 °C for 3 h increased the reaction of the amine group with the aldehyde group of cinnamaldehyde via Schiff base and Michael addition, lowering the GCB film’s water solubility. The addition of BC to gelatin increased the composite film’s tensile strength and decreased its water vapor permeability. The GCB film was nontoxic to L929 cells and possessed significant antibacterial action against *E. coli* and *S. aureus* [156].

He et al. developed a coating material containing CMC and CNC with immobilized AgNPs (CNC@AgNPs) in varying proportions. Compared to uncoated paper, CMC/CNC@AgNPs showed better tensile strength, lower water vapor and air permeability, and greater antibacterial activity against *E. coli* and *S. aureus*. Furthermore, due to the immobilization effect of AgNPs on CNC, the release rate of AgNPs from the coated paper was greatly decreased. When strawberries were packaged using CMC/CNC@AgNPs-coated paper in ambient conditions, strawberries preserved better shape than unpackaged strawberries, and the shelf life was extended to seven days [157].

An antibacterial composite film based on sodium alginate (SA)/CNF containing peanut red skin extract (PSE) was created by crosslinking with Ca^2+^. The results showed that the SA/CNF/Ca^2+^/PSE (SCCP) film had high mechanical strength, great water resistance, and outstanding UV barrier properties. The films had a higher radical scavenging activity in the ABTS assay than the DPPH assay, especially in the presence of 10% ethanol; the maximal ABTS scavenging activity was 99.28%. When the film was applied to pack fruits, the weight loss of fruits was lower for the SCCP film than for the control group. Furthermore, both gram-negative and gram-positive bacteria were successfully prohibited [158].

### 5.3. Intelligent Packaging

Compared to traditional packaging materials, intelligent packaging has attracted great interest in recent years, and the research mainly focuses on environmentally sensitive materials to indicate the food quality changes.

**Figure 8:**
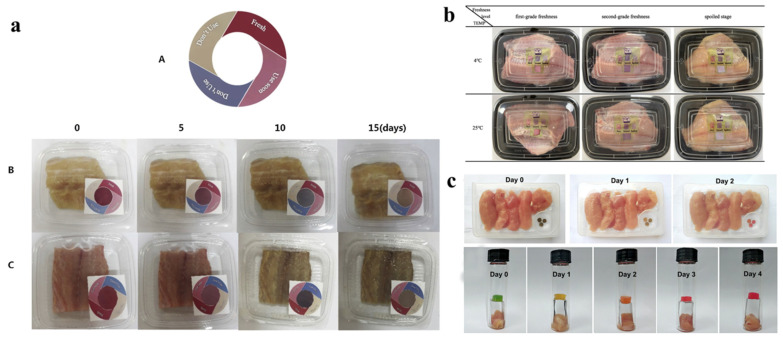
Application of nanocellulose indicator for food freshness monitoring: (**a**) fish, reprinted with permission from [136], 2019, Elsevier; (**b**) tilapia fillets, reprinted with permission from [167], 2022, Elsevier; (**c**) chicken breast, reprinted with permission from [135], 2020, Elsevier.

Moradi et al. developed a unique intelligent colorimetric marker by employing anthocyanins from black carrots and BC nanofibers to check the freshness of rainbow trout and carp slices. Anthocyanin-BC composite film could detect pH change in the packaging materials as storage time increases, and the indicating label changed color correspondingly (Figure 8a). By comparing the label color to the standard color, consumers may visually determine the freshness of fish. The indicator is simple to make, inexpensive, ecologically acceptable materials, and easy to use, thus showing great potential [136].

Shi et al. prepared an intelligent pH-sensitive membrane by combining cyanidin-3-glucoside (C3G) and BC, tested as a tilapia freshness indicator. The results revealed that when BC’s crystallinity increased, the mechanical characteristics of C3G films increased dramatically. BC-C3G films were enhanced in terms of crystallinity and transmittance. Naked eyes can clearly see the color changes of BC-C3G films throughout the freshness monitoring process (Figure 8b), and it has a dependable color response (ΔE) and high sensitivity to TVB-N and TAC changes [166].

As a colorimetric freshness indicator for detecting the freshness of chicken breast, a sugarcane bagasse nanocellulose-based hydrogel was produced. In this process, the nanocellulose was cross-linked by Zn^2+^ to obtain a robust self-standing hydrogel. The pH-responsive dyes (bromothymol blue/methyl red) were incorporated into the hydrogel, which changed color depending on the freshness of the chicken sample. Since CO_2_ levels rose with chicken deterioration due to microorganisms’ growth, the indicator hydrogel’s optical color changed from green to red on the third day (Figure 8c), indicating that the bacterial counts (CFU/g) had exceeded the acceptable limit for human intake. This innovative colorimetric freshness indicator produced with a nanocellulose hydrogel responds quickly to chicken deterioration and is intended to make bagasse nanocellulose more useful as a value-added material in smart packaging [135].

### 5.4. Preservation

The shelf life of food is important since the food products need a few days or even several months until they are delivered to the customers. In order to preserve the food better and extend the shelf life of the products, some kinds of effective packaging are vital.

CNF composite films containing glucose-derived carbon dots (GCD) and N-functionalized GCD (NGCD) were prepared by Ezati et al. The results showed that GCD and NGCD could effectively block the ultraviolet radiation and increase the water vapor permeability of the membrane without affecting the mechanical properties. At the same time, the composite films were resistant to oxidation, with a 99% ABTS and 80–85% DPPH free radical scavenging rate. When these films were applied to citrus or strawberry, they could effectively inhibit the growth of fungi and prolong their shelf life by 2–10 days. In addition, they reduced the weight loss caused by transportation and storage, thus ensuring the freshness of food and improving the economic benefits [166].

A new technique, coaxial 3D printing, was used to create cellulose nanofibers (CNF)-based labels with dual functionalities of fruit freshness preservation and visual monitoring. The shell of fibers was created with CNF-based ink containing blueberry anthocyanin, which had excellent formability and printing fidelity, as well as sensitive visual pH-responsiveness for freshness monitoring. Chitosan containing 1-methylcyclopropene (1-MCP) was injected into hollow microchannels of fibers, where 1-MCP was trapped by the electrostatic effect of chitosan and CNF. The 3D printed labels extended the shelf life of litchis by 6 days while also sensibly indicating variations in freshness, as proven by Headspace-Gas Chromatography-Ion Mobility Spectrometry [168].

## 6. Industrialization of Nanocellulose Production Worldwide

In recent years, the production of nanocellulose from biomass resources has become a hot topic, and intensive investigations have been carried out worldwide. Under this driving force, nanocellulose production has gradually developed from lab-scale to pilot- or even industrial-scale. In Table 6, we summarized the representative companies and institutes producing nanocellulose and pushing the products into markets. It can be seen that the pilot and industrial production lines of nanocellulose are currently mainly located in developed countries, such as the United States, Canada, Japan, Sweden, Finland, etc. As a representative enterprise in the preparation of CNC, CellForce in Canada developed a pilot production line based on sulfuric acid hydrolysis to prepare CNC in 2012, with a production capacity of 1 ton per day. In 2012, the U.S. Forest Service started up the first nanocellulose production plant in the United States in Wisconsin, mainly based on the sulfuric acid hydrolysis method to prepare CNC (10 kg/d) and the grinding method to prepare CNF (1000 kg/d) [169]. In 2014, the University of Maine built up a CNF pilot production line based on the mechanical refining method, with a production capacity of 1 ton per day. In April 2015, American Process realized the commercial production of nanocellulose based on the AVAP method with a production capacity of 1 ton per day, and the nanocellulose products show controllable morphology and surface hydrophilicity and hydrophobicity. It produced lignin-containing nanocellulose, which can realize the reinforcement and filling of plastics [170]. Founded in 2016, Cellulose Lab is a Canadian company providing many different types of nanocellulose products, including CNC, CNF, and BC, and it has become one of the top suppliers of cellulose nanomaterials in the world [171]. Founded in 2015, FineCell in Sweden has invented an oxalic acid-based technology to prepare a nanocellulose product in dry powder, making it easier to be used by mixing with plastics [172]. In 2019, Ocean TuniCell AS in Norway successfully produced tunicate nanocellulose on a large-scale based on microfluidization combined with different pretreatment technologies, such as TEMPO-mediated oxidation, carboxymethylation, and enzymatic treatment. Right now, it has commercialized this unique animal nanocellulose by developing them in to TUNICELL 3D-bioinks [173].

Over the last decades, the industrialization of nanocellulose showed the greatest progress in Japan. Especially after the development of TEMPO oxidation method by Professor Akira Isogai from the University of Tokyo, many Japanese companies have put a lot of effort into commercializing this functionalized nanocellulose [174]. For example, Nippon Paper has begun to build production lines based on this method, with a designed capacity of 500 tons CNF/year [175]. In January 2017, Oji Paper announced that they have started producing CNF with high viscosity and thixotropy based on phosphoric acid pretreatment, followed by mechanical treatment, with a production capacity of 40 tons of CNF per year. Regarding the application of nanocellulose, Oji Paper and Mitsubishi Chemical jointly launched a commercialized nanocellulose (CNF) film in 2013, which can be used to manufacture large-scale displays and solar cells. Oji Paper has also taken advantage of the dense characteristics of CNF and cooperated with Nikko Chemicals to develop its application in cosmetics. Mitsubishi Pencil used the thixotropic properties of CNF as a tackifier for ink, and successfully developed a gel ink ballpoint pen with good thixotropy. Its ink viscosity during writing was reduced by about 50% compared with traditional products, and this product was successfully released in the Japanese domestic market in May 2017 [176]. In addition, nanocellulose also shows great potential in reinforced composite materials. For example, Professor Hiroyuki Kono from Kyoto University has developed a method to prepare a nanocellulose-reinforced resin, and its purpose is to use nanocellulose to reinforce resin materials and use them in automobiles. Since nanocellulose is a light-weight material, its addition could reduce the weight of the car and thus reduce fuel consumption, further promoting the emission reduction of carbon dioxide [177].

The industrialization of nanocellulose also started quite early in Nordic countries such as Sweden and Finland. For example, INNVENTIA is dedicated to promoting the commercialization of CNF and introduced the nanocellulose into mobile phones in February 2011. A pilot-scale production line with a production capacity of 100 kg per day CNF was also manufactured by INNVENTIA [178]. StoraEnso in Imatra and UPM in Lappeenranta are two major Finnish companies who are engaged in the research of microfibrillated cellulose (MFC). In addition, VTT and Aalto University have developed continuous film preparation based on CNF-based plastic materials for food packaging [179].

Although the abovementioned companies have successfully industrialized the nanocellulose production to some extent, these production lines are still mainly based on the sulfuric acid hydrolysis method, TEMPO oxidation method, and mechanical method, and there are still many persistent issues, such as the difficult recovery of inorganic acid, large water consumption, expensive catalyst, or high energy consumption. Recent studies proposed that the AVAP process is sustainable and the chemicals can be recovered; however, due to the use of sulfur dioxide, the entire system needs very high airtightness [180]. Therefore, green, efficient, and sustainable methods for preparing nanocellulose on an industrial scale still require further efforts by researchers.

## 7. Conclusions and Outlook

With the global shortage of petrochemical resources, climate warming, and environmental pollution, people pay more and more attention to how to reduce energy consumption, reasonably allocate nonrenewable resources, expand the use of renewable resources, and take the “green” road in line with the “concept of ecological civilization in the new era”. Nanocellulose and its derived nanocomposite have become a research hotspot in food packaging because of its many excellent properties, including high strength, large specific surface area, excellent barrier property, and good biocompatibility, safety, nontoxicity, and degradability. In food packaging materials, nanocellulose-based nanocomposites can be used as fresh-keeping and antibacterial packaging materials, smart packaging materials, and high-barrier packaging materials, showing the high application potential of nanocellulose-based composites. Therefore, as a kind of renewable and environmentally friendly packaging material, nanocellulose-based composites improve the safety and quality of food and are one of the important directions to realize the development of an “environment-friendly industry” in the food industry in the future.

This review describes the inter-relation of cellulose chemical structure and its source, and their different physicochemical properties are discussed. Though cellulose extracted from plants is the most investigated, the cellulose purified from bacteria and animals with unique structural characteristics has raised increasing interest. To date, enzymatic hydrolysis, TEMPO-oxidation, and carboxymethylation are widely utilized pretreatments to help defibrillation of cellulose during processing into nanocellulose, which not only helps reduce the energy consumption but also provides extra functional groups for the final products. Acid hydrolysis, including both mineral and organic acids, could remove amorphous regions, resulting in cellulose nanocrystal (CNC), though the highly corrosive conditions and the low yield of CNC are persistent issues. Through mechanical treatments, such as refining, homogenization, microfluidization, sonification, ball milling, and the aqueous counter collision (ACC) method, cellulose nanofibrils (CNF) could be produced, but the extremely high energy input prohibits the commercialization of these techniques. Bacterial cellulose (BC), a unique bacteria-derived nanocellulose, has recently gained numerous research interests. Its higher aspect ratio and larger diameter make it a promising material for food packaging applications. In order to facilitate the application of nanocellulose in food packaging, it has always been processed to different forms of materials, such as film, gel, coating membrane, and emulsions, by various fabrication technologies, including solution casting, Layer-by-Layer (LBL) assembly, extrusion, coating, gel-forming, spray drying, electrostatic spinning, adsorption, etc. Thanks to the nontoxicity, good biodegradability and biocompatibility, high aspect ratio, low thermal expansion coefficient, excellent mechanical strength, and unique optical properties, nanocellulose-based food packaging materials have been widely applied to pack fruits, meat products, instant foods, dairy products, and beverages. Since nanocellulose and the functional fillers incorporated into the cellulose-based nanocomposites impart the materials’ excellent barrier and mechanical properties, antibacterial activity, and stimuli-responsive performance, they have greatly improved the quality stability and shelf life of foods.

So as to provide sufficient nanocellulose products for green food packaging, many companies in Europe, Africa, and Asia have been pushing the lab-scale production of nanocellulose for a pilot- or even industrial-scale production. However, there are still several persistent issues that should be overcome in the near future to realize the successful commercialization of nanocellulose-based composites as food packaging materials.

1. Expansion of the cellulose sources to other biomass besides the traditional raw materials, such as tunicate and BC, for higher quality nanocellulose to develop many advanced applications. Until now, the nanocellulose on the market is dominantly produced from wood and other plant-based sources. Though several works of research focused on the nanocellulose preparation from tunicate and BC and demonstrated their better performance than the woody nanocellulose, further investigation on the preparation–characterization–performance correlation of this specific nanocellulose is necessary.
2. Development of facial, cost-effective, efficient, and environment-friendly nanocellulose extraction method. Though several novel extraction methods have been developed, sulfuric acid hydrolysis and mechanical refining are still the most widely used ones. However, the harsh acid hydrolysis, high water consumption, huge amount of polluted wastewater, intense energy consumption, and low yield greatly prohibited industrially feasible nanocellulose production. Therefore, more efforts should be put into developing new nanocellulose preparation methods, such as organic acid-based methods, which already showed potential to be a green approach to preparing functionalized nanocellulose.
3. Development of new cellulose nanocomposite fabrication approaches. In the lab, the solution casting method is still widely used for research purposes, which is unsuitable for industrial production. In the pilot scale, extrusion is used, though it is not a perfect method since nanocellulose is always dispersed in water, which negatively affects the extrusion performance. Therefore, developing a scalable strategy to prepare nanocellulose-based composites for food packaging materials is vital.
4. Improvement of the performance of nanocellulose-based composites as packaging materials. As discussed above, the ideal food packaging materials require UV-proof, gas and vapor barrier properties, excellent mechanical force, and good hydrophobicity. Especially for the last one, new strategies need to be developed to alter the hygroscopic nature of nanocellulose and enhance the wet strength, thus making its applications more practical in daily life. For example, esterification as a pretreatment or coating with natural wax seems suitable to fulfil this purpose.
5. Development of nanocellulose-based intelligent packaging materials. Currently, achieving the cellulose nanocomposites’ responsive properties is mainly realized by incorporating various organic and inorganic fillers. However, the release and migration of functional fillers and their potential health risks have not been comprehensively evaluated. Future studies should not only focus on the safety issue of the nanocellulose itself but also on the functional fillers used.
6. Design of the food-specific, nanocellulose-based packaging materials. Though many research works generally focused on the preparation and properties of packaging materials, they paid little attention to the interaction between the materials and the food, and even ignored various aspects which would influence the application of the materials. For example, the influence of the environmental conditions on the quality change of both food products and the packaging materials should be investigated to prove the feasibility and suitability of the packaging materials for the specific food.

## Figures and Tables

**Table 1:** Examples of the geometrical dimensions and crystallinity index of nanocellulose from various sources.

Source	Length (nm)	Width (nm)	Crystallinity Index (%)	References
Softwood	483 ± 232	4.1 ± 1.2	/	[49]
Cotton linter	177	12	90.45	[44]
Algae	315 ± 30	9 ± 3	81	[50]
Bacteria	/	64.6 ± 15.3	90.3	[51]
Tunicate	2100 ± 700	8.7 ± 2.4	/	[49]

**Table 3:** Methods for the preparation of BC.

Culture Medium	Bacteria	Conditions	Characteristics	References
Wine pomace	*Komagataeibacter rhaeticus* K15	30 °C, 10 days	Yield 1.95 ± 0.22 g/L, nanocellulose concentration 91.67 ± 2.76%, crystallinity index 90.61%, diameter range 30–130 nm	[113]
HS	*Taonella mepensis*	30 °C, 7 days	Yield 2.472 g/L, crystallinity index 90.3%, average width 64.6 ± 15.3 nm	[51]
ST	*Taonella mepensis*	30 °C, 7 days	Yield 1.784 g/L, crystallinity index 82.8%, average width 53.3 ± 20.1 nm	[51]
Sugarcane bagasse	*Komagateibacter xylinus*	28 °C, 9 days	Fiber diameter 47 ± 10 nm, crystallinity index 79%, water content 99.43 ± 0.03%	[114]

**Table 4:** Nanocellulose-based composites for food packaging applications.

Cellulose Component	Other Components	Method	Performance	References
CNC	Chitosan	Solution casting	The tensile strength and Young’s modulus of the film have been increased by 39% and 78%, respectively. Water solubility has been reduced by 26.5–35.7%, with good UV resistance and water repellency.	[116]
CNF	Polyurethane(PU), quaternized chitosan (QCS) and negatively charged phosphotungstic acid	Layer-by-Layer assembly	The conductivity of hydroxide reached 14.3 mS/cm at 80 °C and lasted for more than one month.	[117]
CNC	Polylactic acid (PLA)	Extrusion	Improved processability, melt strength, and rheological properties. Good performance in storing oil-based and dairy products can prolong their shelf life.	[118]
CNC	Palm oil/water	Emulsions	Checking whether food is spoiled or not.	[119]
BC	Protein nanoparticles	Hydrogel	Good wettability, interfacial adsorption capacity, and higher antioxidant property.	[120]
CNF	Polylactic acid (PLA), Chitosan, rosin	Spray drying	Great antibacterial effect and increased elasticity and water vapor permeability.	[121]
CNC	Chitosan, polyvinyl alcohol	Electrospinning	Preventing the growth of pathogenic bacteria	[122]
CNF	Polyvinyl alcohol (PVA), Silver Nanoparticles	Electrospinning	Good antibacterial activity against *Staphylococcus aureus, E. coli*, and *P. aeruginosa*.	[123]
BC	Bovine Lactoferrin (BLF)	Adsorption	Strong bactericidal effect on *E. coli* and *Staphylococcus aureus*.	[124]
CNC	Polyethylene glycol, algal bile protein	Adsorption	Improved protein stability.	[125]

**Table 5:** Application and performance of nanocellulose-based food packaging materials.

Composition	Performance	References
CS/carboxymethylated nanocellulose	Increased resistance to grease, oil, water, air, and water vapor. Good mechanical characteristics and enhanced antibacterial activity against both *E. coli* and *Staphylococcus aureus*.	[154]
Corn starch/BC	Improved barrier to water vapor and oxygen.	[155]
Gelatin/BC/cinnamaldehyde	Increased tensile strength and lower water vapor permeability. Inhibited against *Staphylococcus aureus* and *E. coli* germs.	[156]
CMC/CNC/AgNPs	Excellent mechanical strength, water vapor and air barrier characteristics, and antibacterial activities.	[157]
SA/CNF/Ca^2+^/PSE	High strength, good water resistance, excellent ultraviolet barrier performance, and significant antibacterial effects against both gram-negative and gram-positive bacteria.	[158]
Anthocyanins/BC	Consumers can judge the freshness of fish by comparing the label color with the standard color.	[136]
EAE/BC	The fabricated BC-EAE indicator responded to pH by changing color from red to yellow over the pH range of 2–12.	[159]
AgNPs@CS-1:1	The storage time of strawberries packaged by AgNPs@CS-1:1 was extended to 12 days without microbial invasion.	[160]
SA/Carboxymethylated nanocellulose/SOWEs	Great effect on controlling browning index in fresh-cut apple and potato over the storage of 12 days and 5 days.	[161]
CS/guar gum/walnut green husk extract	Good performance in reducing firmness, weight loss, total soluble solids, and inhibiting browning and microbial growth of fresh-cut apples.	[162]

**Table 6:** Global nanocellulose production facilities with their products and capacities.

Company	Country	Method	Products	Production Capacity
INNVENTIA	Sweden	Enzymatic and microfluidizer	CNF	100 kg/d
Nippon paper	Japan	TEMPO oxidation and mechanical defibrillation	CNF	150 kg/d
Stora Enso	Sweden	Enzymatic and mechanical defibrillation	MFC	n.a.
CellForce	Canada	Sulfuric acid hydrolysis	CNC	1000 kg/d
U.S. Forest Service	U.S.	Sulfuric acid hydrolysis and drinder	CNC and CNF	CNC (10 kg/d) and CNF (1000 kg/d)
Cellulose Lab	Canada	n.a.	CNC, CNF and BC	n.a.
American Process	U.S.	AVAP	CNF	1000 kg/d
University of Maine	U.S.	Mechanical refining method	CNF	1000 kg/d
VTT	Finland	Enzymatic pretreated with Masuko grinder	CNF	15 kg/d
FineCell	Sweden	Oxalic acid hydrolysis	CNF	n.a.
Ocean TuniCell AS	Norway	Enzymatic, TEMPO oxidation, carboxylmethylation, and microfluidizer	Tunicate CNF	n.a.

## Data Availability

Not applicable.
